# Septin clearance from the division site triggers cytokinesis in budding yeast

**DOI:** 10.15698/mic2019.06.681

**Published:** 2019-05-22

**Authors:** Davide Tamborrini, Simonetta Piatti

**Affiliations:** 1 Centre de Recherche en Biologie Cellulaire de Montpellier 1919 Route de Mende, 34293 Montpellier, France.

**Keywords:** septins, cytokinesis, actomyosin ring, Mitotic Exit Network

## Abstract

In many eukaryotic cells cytokinesis involves a contractile actomyosin ring (CAR) that drives cleavage furrow ingression. What triggers CAR constriction at a precise time of the cell cycle and how constriction is coupled to chromosome segregation are fundamental questions. In the budding yeast *Saccharomyces cerevisiae*, CAR assembly strictly requires a rigid septin collar that forms at the bud neck early during the cell cycle. At the time of cytokinesis, a sudden remodelling of the septin collar occurs, leading to its splitting into two separate rings that sandwich the CAR. We have shown that septin displacement during splitting is an essential prerequisite for CAR constriction [Tamborrini *et al*., Nat Commun. 9(1):4308]. Thus, cytokinesis in budding yeast is a two-step mechanism: during the first step, the septin collar organizes the assembly of the cytokinetic machinery at the right place while restraining CAR-driven membrane ingression; during the second step, a confined eviction of septins from the division site during septin ring splitting triggers CAR constriction. Our data further indicate that septin ring splitting is prompted by the Mitotic Exit Network (MEN), and in particular by its downstream phosphatase Cdc14, independently of its mitotic exit function. Surprisingly, MEN signalling at spindle pole bodies (SPBs) is critical for septin ring splitting and cytokinesis. Ubiquitination of the MEN anchor at SPBs by the Dma1/2 ubiquitin ligase attenuates MEN signalling and could have a decisive role in coupling cytokinesis to chromosome and organelle segregation. Altogether, our data emphasize the importance of septin ring splitting, which has been mysterious so far, and highlight a novel mechanism to prevent CAR constriction and cytokinesis in unpropitious conditions.

Cytokinesis is the fundamental process that closes the cell cycle by physically separating daughter cells. In eukaryotic cells cytokinesis is temporally and spatially controlled to ensure the balanced partitioning of chromosomes and organelles, thereby contributing to the stability of a cell lineage. Additionally, the position of the cleavage furrow is a primary determinant for asymmetric versus symmetric cell division, which in turn profoundly affects cell identity and fate. Not surprisingly, cytokinesis failure has been linked to aneuploidy and cancer.

In animal cells and fungi the cytokinetic machinery comprises a contractile actomyosin ring (CAR) and septin assemblies at the division site. Both structures interact tightly with the plasma membrane, but while CAR constriction is known to drive membrane ingression, the role of septins in cytokinesis remains quite elusive. Fly and human septins have been recently shown to bundle and curve actin filaments into rings, thereby likely participating to CAR formation. In contrast, budding yeast septins are unable to bundle actin filaments, yet they are essential for CAR assembly and cytokinesis. Current models envision that budding yeast septins scaffold the cytokinetic machinery at the bud neck (the constriction between mother and daughter cell where cytokinesis occurs). In *S. cerevisiae* septins form a ring at this location already in late G1, before bud emergence. At the onset of S phase, the septin ring is expanded into a rigid collar that persists throughout most of the cell cycle and recruits many cytokinetic factors to the bud neck, thus supporting its scaffolding function. At the end of mitosis, however, the septin collar undergoes a sudden and spectacular rearrangement leading to two split rings that sandwich the CAR. Our recent data indicate that CAR constriction shortly follows septin ring splitting (within 4-5 minutes), suggesting that the intact septin collar might actually restrain CAR constriction. However, a clear assessment of the physiological function of septin ring splitting has been hampered by the absence of mutants specifically defective in this process.

**Figure 1:**
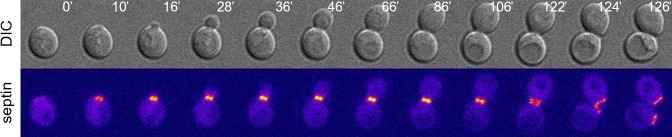
FIGURE 1: The budding yeast septin collar during the cell cycle. Wild type cells expressing the septin Cdc12 tagged with GFP were imaged at 30°C and Z-stacks were max-projected and pseudocoloured “fire” with Image J. Septins appear at the presumptive bud site in unbudded cells (t=10') and the septin collar splits in late mitosis (t=122', white arrow). DIC: differential interference contrast.

Septin ring splitting requires an essential Hippo-like kinase cascade named Mitotic Exit Network (MEN), which triggers mitotic exit (i.e. inhibition of mitotic CDKs) and cytokinesis. The role of MEN in mitotic exit is mediated by its downstream effector, i.e. the Cdc14 phosphatase that dephosphorylates mitotic CDK substrates and concomitantly inactivates mitotic CDKs. In particular, MEN liberates Cdc14 from its nucleolar anchor Net1 to allow it reach its nuclear and cytoplasmic targets. While mitotic exit is an essential prerequisite for cytokinesis, MEN factors are thought to participate to cytokinesis also independently of Cdc14 nucleolar release. In our paper, we inactivated MEN factors under conditions that are permissive for Cdc14 nucleolar release to assess their possible direct role in septin ring splitting. Through this analysis we showed that a subset of MEN factors is required for septin ring splitting and CAR constriction independently of Cdc14 release from the nucleolus and mitotic exit. Furthermore, we found that overexpression of the E3 ubiquitin ligase Dma2 inhibits MEN signaling and specifically interferes with MEN-induced septin ring splitting and CAR constriction without preventing mitotic exit.

This latter experimental set-up allowed us to test rigorously the importance of septin ring splitting for CAR constriction. Remarkably, we showed that septin clearance from the division site is sufficient to trigger CAR constriction and cytokinesis upon Dma2 overexpression. These results have two main implications: first, septin ring splitting at the bud neck not only precedes, but is a critical prerequisite for CAR constriction. Second, this essential displacement of septins from the division site can be achieved through either splitting or complete clearance/disassembly. Thus, although septins are needed for the assembly of the cytokinetic machinery and the CAR before cytokinesis, at cytokinesis they need to be removed from the division site to allow CAR constriction and cleavage furrow ingression. This two-step mechanism provides an intrinsic safeguard device that imposes the correct temporal order of cytokinesis events. Other organisms might exploit a similar safety device. For instance, in *Schizosaccharomyces pombe*, where the septin ring at the division site is dispensable for CAR assembly, the septin ring also splits in two before cytokinesis, raising the possibility that the septin ring might restrain CAR constriction also in fission yeast.

How exactly the septin ring holds back CAR constriction in yeast is a crucial question to be addressed in the future. One possibility is that the septin collar acts as a physical constraint by either preventing proper contacts between CAR and plasma membrane or harnessing the CAR in a non-constrictable arrangement. Alternatively, septins might hinder CAR access to regulators of constriction, such as the Rho1/RhoA GTPase. Interestingly, yeast septins suppress the activity of the Rho GTPase Cdc42 during budding, raising the possibility that a similar mechanism might operate at cytokinesis.

In our experimental framework septin splitting and disappearance both trigger CAR constriction at cytokinesis. So why did yeast cells choose to maintain the two split septin rings after cytokinesis instead of dismantling them altogether? We propose that the aim of preserving split septin rings during and immediately after cytokinesis is to provide a positional cue at the membrane of haploid cells for the establishment of cell polarity in next cell cycle. Indeed, cell polarization at the presumptive bud site follows a stereotypical pattern that is different in haploid and diploid cells (i.e. axial vs. bipolar), with the axial budding pattern involving septins.

According to our data, the phenotype of *DMA2*-overexpressing cells is remarkably similar to that of MEN mutants forced to exit mitosis upon loosening the interaction between the Cdc14 phosphatase and its nuclear anchor Net1: in both cases cells can exit mitosis, enter a new round of budding in the following cell cycle and assemble a new septin ring before splitting/disassembly of the old septin ring. These results raised the possibility that Dma2 could be a MEN inhibitor. We confirmed this hypothesis by showing that Dma2 binds to and ubiquitylates the MEN scaffold Nud1 at spindle pole bodies (SPBs, i.e. the yeast microtubule-organising centers). Nud1 is essential for MEN signaling by acting as a platform that activates MEN components in a sequential manner. We showed that Nud1 ubiquitylation by Dma2 weakens the recruitment of MEN factors (including Cdc14 itself) to SPBs and interferes mildly with mitotic exit and conspicuously with septin ring splitting and cytokinesis. Strikingly, artificial anchoring of Cdc14 at SPBs largely rescues the cytokinesis defects of *DMA2*-overexpressing cells, suggesting that SPB-localized Cdc14 is crucial for septin ring splitting. Thus, not only Cdc14 must be released from the nucleolus by the MEN cascade, but also needs to reach the SPB to carry out its cytokinetic functions. We think that the physiological role of Nud1 ubiquitination is to silence MEN signaling after cytokinesis has been accomplished. However, the same process could be beneficial under different detrimental conditions, e.g. to prevent cytokinesis before proper chromosome or organelle segregation is complete. Accordingly, we previously showed that deletion of *DMA2* and its paralog *DMA1* leads to unscheduled mitotic exit and cytokinesis upon spindle mispositioning.

**Figure 2:**
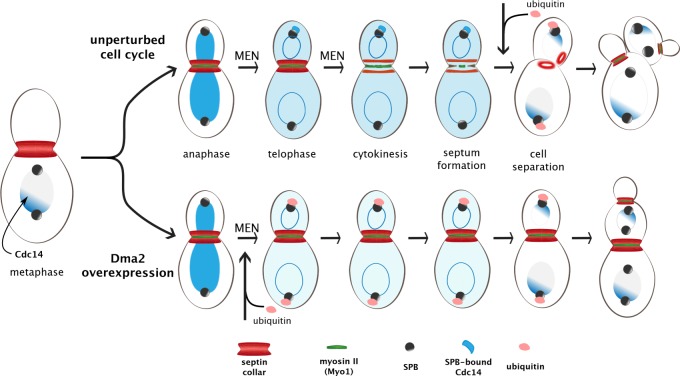
FIGURE 2: Ubiquitylation-mediated control of septin ring splitting and cytokinesis. At the onset of anaphase, the Cdc14 phosphatase (in blue) is released from the nucleolus and diffuses to the nucleus. In telophase, its full release to the cytoplasm prompted by the Mitotic Exit Network (MEN) enables it to reach the spindle pole body (SPB; predominantly the bud-directed SPB) and later on also the bud neck (not depicted). At this stage MEN promotes also septins ring splitting, which in turn allows constriction of the Contractile Actomyosin Ring (CAR) and septum formation. Notice that the myosin II (Myo1) component of the CAR appears at the bud neck in late G1 along with septins, while actin (not depicted) is recruited only in late mitosis in a MEN-independent manner. Once cytokinesis is accomplished, the ubiquitin ligases Dma1/2 ubiquitinate the MEN scaffold Nud1 and shutdown MEN signalling at the SPB. Cdc14 is trapped back in the nucleolus and the two daughter cells start a new cell cycle. Upon Dma2 overexpression (bottom), precocious ubiquitylation of Nud1 hinders Cdc14 recruitment to the SPB, thereby preventing septin ring splitting and CAR constriction. Upon cytokinesis failure daughter cells start nevertheless a new cell cycle, forming new septin rings and new buds, resulting in chained cells with a shared cytoplasm.

How does MEN signaling and Cdc14 at SPBs promote septin ring splitting at the bud neck? We speculate that in its cell cycle journey Cdc14 needs to pass by the SPBs to be able to reach the bud neck, where it transiently appears shortly before septin ring splitting. At the bud neck Cdc14 would then dephosphorylate critical substrates that trigger septin displacement. It is worth noticing that Cdc14's requirement for mitotic exit seems peculiar to budding yeast, while its involvement in cytokinesis is conserved. Whether the role of Cdc14 in the control of cytokinesis in other organisms is linked to regulation of septin dynamics remains to be established. Likewise, how exactly Cdc14 triggers septin ring splitting in budding yeast is an exciting subject for future studies. The identification of its targets will be key to further dissect the mechanistic details underlying this process.

